# The Diamond Jubilee and the Hospitals

**Published:** 1898-01-22

**Authors:** 


### THE INSTITUTIONAL WORKSHOP.

### THE DIAMOND JUBILEE AND THE HOSPITALS.

### INTRODUCTORY.

The Diamond Jubilee promises to produce something like a comedy in hospital affairs. The origin of the comedy is the mistaken idea which pervades many minds to the effect that, to get money from the British public, it is necessary to adopt the tactics of the street contortionist, who lives by begging. It is popularly supposed that the more agonising the spectacle the larger becomes the beggar's revenue. So the absurd fallacy has grown up in the Metropolis of the Empire that to secure funds for charitable purposes it is necessary to overstate the case and exaggerate the figures. In plain English those who resort to these practices are nothing more nor less than mendacious mendicants of the worst class, and it is time that a strong protest should be made and a note of warning sounded before permanent injury is done to our excellent system of voluntary contributions. The Prince of Wales's Fund is a case in point. The Council have endeavoured, after careful inquiries and the receipt of attested statements from the larger hospitals, to apportion their income on a plan which shall produce permanent benefit to the sick poor of the metropolis. With a wisdom which circumstances have already justified, the grants from income have been made on certain conditions, and it is reasonable to assume that if those conditions are not fulfilled the grants will be subject to readjustment. The object aimed at by the Prince of Wales's Fund appears to be to help each hospital by relieving it of certain disabilities which have been put forward as a special reason why a generous public should give largely to its funds. This has been done, no doubt, in view of the Prince of Wales's letter of February last, to the effect that if the hospitals are to be saved from State or parochial aid their financial condition must be assured.

The aim of the Prince's Commemoration Fund was "to secure for these necessary institutions sufficient and permanent support." The principle underlying the Prince of Wales's argument was, that the hospitals should be placed upon a sufficiently wide and permanent financial basis. To effect this the revenue of the Fund has been distributed in the directions we have indicated. If the object aimed at is ever to be attained, it follows that the hospitals on their part must co-operate by determining not to incur extraneous expenditure which they would not have thought of incurring had they not received a large permanent addition to their income through the Prince of Wales's Fund. It seems necessary to point out this patent fact, for, from an official communication issued to the Press by Guy's Hospital, it would appear, that despite the fact that a grant has been made from the Prince of Wales's Fund of an amount amply sufficient to defray the cost of the 114 vacant beds which it is desired to restore to the use of the poor, the Treasurer of Guy's Hospital declares: "It should be understood that the annual expenditure necessary to maintain the reopened wards will exceed the grant from the Fund, liberal though it is; and, owing to the increased number of patients, it will be necessary to undertake very extensive structural alterations in the laundry and other departments." The extra income allocated to Guy's Hospital by the Prince's Fund to enable the Governors to reopen the 114 beds is stated to be £6,600. This sum should be amply sufficient to defray all the costs of maintaining the 100 beds, which it is presumed will be about the number constantly occupied, seeing that they are a portion only of a larger number in a hospital the upkeep of which has been provided in past years, although the wards about to be reopened were closed to poor patients. If a hospital like the Poplar Hospital, with 67 beds in the main building and six beds in a separate block, making 73 beds in all, costs altogether but £5,368 per annum, it must be apparent to hospital managers of experience that the sum apportioned to Guy's Hospital should be amply sufficient to defray all the expenditure to which the authorities of Guy's Hospital will be put by opening the vacant wards and maintaining therein 100 beds. We are confident this is a view which business men who support hospitals are likely to take; and we think it is a little unfortunate that Guy's Hospital, having regard to the extraordinary liberality manifested towards it by the public during the last three years, should have put forward the statement to which we refer with the apparent view of inducing the public to believe that they must still put their hands into their pockets and contribute liberally to the support of the reopened wards, when in practice the necessity for any such contributions ought not to exist as a matter of fact.

Our point is that those hospital managers, who fail to recognise that the attempt to place them upon a permanent financial basis must be reciprocated, will soon find, if they fail to act upon this principle, that their revenue from voluntary sources will tend to diminish, and not to increase. The increasing interest taken in hospitals and their management in recent years has educated the public to understand better what are the actual needs of each institution. Those institutions which show that the management recognises this fact, and is loyally acting upon it, will win public confidence and secure adequate funds. Those institutions, on the other hand, which accept the permanent addition to their income derived from the Prince's Fund in an apologetic spirit, and which at once set to work to explain away the advantages of such splendid pecuniary assistance, deserve, and we have no doubt will receive, a severe lesson at the hands of the public who support hospitals. There must, as we have said, be finality in expenditure, and for a hospital receiving a permanent addition of several thousands a year to its income from the Prince of Wales's Fund to calmly issue a statement, which, in expressing gratitude for such generous help, proceeds to explain it away by urging that still larger funds are necessary, owing to the increase which must necessarily arise in the upkeep by providing more accommodation for nurses, or for resident medical officers, or for a separate laundry, or for additional operator theatres, and so forth, will show a lack of worldly wisdom and a departure from a strict regard to the requirements and needs of the moment, which may entail upon them a sharp lesson from the hospital supporters of our great voluntary charities. The first thing that every hospital has to convince the public of is, that its managers welcome the additional revenue, and are determined, now that their resources are increased to an amount which will reduce the annual deficiency to reasonable limits, to set themselves to make good that deficiency in the first instance, and so to place the financial management upon a sound footing, and to prevent once and for all the issue of over-coloured or exaggerated statements, or, indeed, any special appeal at all.

### Factors in the Problem.

On a famous occasion the late Mr. W. H. Smith, when First Lord of the Treasury, said that there was a lying spirit abroad. Unfortunately, philanthropy in this country in our day and generation has attained such gigantic proportions that it has necessarily attracted a large number of people, the least worthy of whom think it justifiable, so long as they raise money for the particular object with which they are connected, to utter reckless statements which in their view will bring in funds to the cause. Signs are not wanting that this policy is to be pursued by certain of the medical institutions in London, and, of course, they are supported in this attitude by the print which ekes out a precarious existence with their assistance. In these circumstances, we propose from time to time, as the data are forthcoming, to publish actual facts, believing that there is no way so effectual in breaking down a bad system as the fullest publicity based upon precise knowledge. Meanwhile, we hope that all persons who have it in their power to give liberally to charity will set their faces like a rock against those institutions which pelt them with harrowing appeals, padded with statements to the effect that they have been seriously injured by the Diamond Jubilee memorials, or that they are starving for want of funds, unless they will first take the trouble to write to the managers of these institutions, to get from them a statement showing the figures on which such a plea is based, and then submit those figures to a competent authority for audit. If the public can once be induced to realise that harrowing appeals of this character may properly be taken as evidence that the management lacks something in business quality and conscientious accuracy, appeal literature will promptly be purged of an abuse which threatens to close the public purse, and so to injure the most worthy and best administered institutions.

We have had a very large and exceptional experience in raising money for charities, and we are convinced that the most successful appeal is the one which contains nothing but audited figures, which is tersely written, and undertakes on the part of the managers to do such and such work, which is urgently required to be done for the less fortunate members of society, provided such and such moneys are supplied by the public. The late Mr. Spurgeon was a power in the charitable world, and he never failed to get all the money he wanted for every object which met with his approval. Mr. Spurgeon's success, apart from his individual gifts, which were considerable, was, as he once told us, due under Providence to the enforcement of a policy which treated the public with entire confidence, and placed upon them the responsibility of either producing the money required, or of leaving the work unaccomplished. When one sees a hospital, directly it has received a splendid gift or a material addition to its income, putting forth a statement which belittles the help which is thus extended to the managers, and urges that further and further expenditure will be necessary, and that there must still be an ever-increasing and ever-constant deficiency, our readers may depend upon it that, however good such a hospital or instititution may be, if this policy is not altered the public will find them out, and funds will gradually fall off. We are only too grateful to know that this is so, and we could wish that its truth could be driven home to the minds and consciences of every hospital committee throughout London, as it has already been driven home to provincial hospital managers, who have largely profited by its enforcement. In any case, whatever the nature of the work undertaken by a particular institution, we do most earnestly urge the public who subscribe to charities not to give in response to whining appeals, which deal mainly with the poverty, the increasing needs, and the large deficiency of the particular charity which desires to secure their gifts.

We believe that when the data are forthcoming it will be found that, so far from injuring any well-managed hospitals, the Diamond Jubilee year has been on the whole one of reasonable prosperity, and that the money received from voluntary contributions is well up to the average. We have been assured by some of the ablest and most intelligent of hospital managers that this is so, and where the contributions have fallen off the cause is probably due to diminished energy on the part of those responsible, who have rested on their oars instead of working at their full strength to replenish the hospital exchequer for 1897.

### Dangerous Ingratitude.

The hospitals have been much before the public during the past twelve months, and many representative and busy men have devoted a large portion of their time to an endeavour to make the Diamond Jubilee year the occasion of permanently helping the hospitals. One of the most notable features of these efforts has been the hostile and ungenerous criticism of a certain class of persons who have never given any time to similar efforts, and who have, with equal probability, never been the means of adding one single pound to the hospital resources. The lines of argument these people adopt is to belittle every effort to help the hospitals collectively, to publish misleading and inaccurate statements about the Hospital Sunday Fund, or the Hospital Saturday Fund, or the Prince of Wales's Fund, and to treat with contempt movements of this kind which collectively hand over to the hospital managers of London a sum which in the whole is not far short of 20 per cent. of their entire revenue. Anyone reading the fulminations to which we refer might conclude that it was penal for public men or women to associate themselves together to help the hospitals as a whole. The argument is that any such efforts interfere with the means of raising revenue which eac institution has to keep going for its maintenance. They altogether ignore the fact that the proportion of people in a large population like that of London who give any regular support, or, indeed, any support at all, to the hospitals is remarkably small. For this reason, instead of these collective efforts interfering with the individual efforts of particular institutions, providing the latter are intelligently and ably administered, they do but enlist the sympathies and attract the gifts of an ever-extending number of persons who would never give at all to the hospitals were it not for the special efforts of the great funds to which we refer. One of the most satisfactory and encouraging signs of the Diamond Jubilee year is the circumstance, that the Prince of Wales's Fund has throughout acted in cordial co-operation with the Hospital Sunday and Saturday Funds, and that every effort has been made to prevent overlapping, and so to extend the area of givers to the hospitals of London.

Another point which seems to be forgotten, and which has probably never been realised by the authors of the unintelligent criticisms we have in view, is that there are certain crops which require time to develop. Any capitalist who puts his money into tea, or coffee, or cinchona knows that for the first few years he must not expect any return upon his capital; that for a few succeeding years he will only get a small return; and then, when the plantations have become established, that he will reap the reward of his enterprise. So in the case of the Prince of Wales's Fund, having regard to the number of hospitals in the metropolis, it was necessary to begin with the larger institutions, some 17 in number, to go thoroughly into their circumstances, and to render them permanent assistance, as the parents of our hospital system and the most important and representative of our medical institutions. An enterprise like that of the Prince of Wales's Fund, if it is ever to be established on an adequate and firm basis, must move slowly at the outset. It was impossible in ten months to do everything, and we are sure that the managers of all hospitals will come to see that the Council have shown the sincerity of their wish to do the work thoroughly by confining themselves in the first year to what was possible in the circumstances. No doubt during 1898 every effort will be made to thoroughly master the condition and merits of each of the London hospitals, whatever its size may be. Meanwhile, every metropolitan hospital receiving a grant from the Hospital Sunday Fund has had that amount doubled by the special grants in honour of Her Majesty's Diamond Jubilee, which they have received from the Prince of Wales's Fund. The awards made are in our view in accordance with the original idea, which is evidently being pursued systematically and carefully, and which will ultimately result, we have little doubt, in placing every reputable metropolitan hospital on a sound financial basis.

